# Red Grape Pomace as a Quality-Modulating Ingredient in Dairy Cattle Salamis

**DOI:** 10.3390/foods15101792

**Published:** 2026-05-19

**Authors:** Gabriele Busetta, Giuseppe Maniaci, Marcella Barbera, Cristina Giosuè, Simone Italia, Daniela Piazzese, Luca Settanni, Marco Alabiso, Raimondo Gaglio

**Affiliations:** 1 Department of Agricultural, Food and Forest Sciences, University of Palermo, Viale delle Scienze, Bldg. 5, 90128 Palermo, Italy; gabriele.busetta@unipa.it (G.B.); giuseppe.maniaci@unipa.it (G.M.); luca.settanni@unipa.it (L.S.); marco.alabiso@unipa.it (M.A.); 2 Department of Earth and Marine Sciences, University of Palermo, Via Archirafi, 22, 90123 Palermo, Italy; marcella.barbera@unipa.it (M.B.); daniela.piazzese@unipa.it (D.P.); 3 Centre for Sustainability and Ecological Transition, University of Palermo, Piazza Marina, 90133 Palermo, Italy; 4 Le Liccumie S.r.l., Contrada Fargione Aggl. Ind., 97015 Modica, Italy; simone.italia@leliccumie.com

**Keywords:** grape pomace powder, Cinisara breed, fermented meat, microbiological safety, physicochemical characteristics, sensory evaluation, consumer acceptability

## Abstract

This study investigated the effects of red grape pomace powder (GPP) on spontaneously fermented salamis produced from the meat of retired cows and young bulls of the Cinisara dairy breed. The use of GPP and meat from these animal categories was motivated by the valorization of low-commercial-value agri-food resources and the enhancement of sustainable local production chains. Plate count analyses showed typical fermentation dynamics, with lactic acid bacteria (LAB), coagulase-negative staphylococci, and yeasts reaching approximately 7 log CFU/g, and confirmed the absence of major foodborne pathogens. Illumina sequencing further characterized the bacterial community, identifying *Latilactobacillus* as the dominant genus at the end of ripening, with relative abundance (RA) of up to 65% in GPP-enriched trials. Physicochemical analyses showed progressive changes during ripening, including weight loss, pH decrease, color development, and increased proteolysis. GPP supplementation contributed to the stabilization of a*, chroma, and hue values, while reducing lightness during ripening. Oxidative stability measurements showed that GPP derived polyphenols effectively limited oxidative reactions, especially secondary lipid oxidation. GPP also modulated the volatile profile by increasing ester formation and introducing plant-derived compounds. Sensory evaluation revealed higher color intensity and aroma in enriched salamis, along with higher bitterness and lower structural homogeneity, especially in those produced from retired cows. Consumer surveys conducted in two retail settings indicated strong interest in this innovation, with over 80% of respondents willing to pay a 10–20% price premium. Overall, GPP emerges as a promising functional ingredient for enhancing, diversifying, and valorizing fermented salamis produced from dairy cattle meat, supporting both product innovation and sustainability-oriented strategies.

## 1. Introduction

Salami is a long-established commercial category of fermented meat products, appreciated for its distinctive sensory attributes, including a complex aromatic profile, firm texture, and broad culinary versatility [1]. In recent years, evolving consumer expectations have driven the meat industry to develop products with enhanced nutritional value and fewer synthetic additives [2]. This demand has stimulated research into functional meat products enriched with plant-derived by-products, which can enhance technological quality and provide bioactive components with potential health benefits [3]. The use of natural ingredients is also consistent with the priorities of the National Recovery and Resilience Plan, which promotes nature-based solutions to support a circular, low impact economy while ensuring public health protection [4,5].

Consequently, plant- and fruit-derived industrial by-products are increasingly used to improve the functional properties of foods that are naturally low in bioactive compounds [6]. At the same time, growing market diversification has generated interest in salamis produced from non-pork species. Although traditionally made from pork, salamis produced from alternative meats are gaining attention due to their distinctive sensory traits and their potential to meet consumers’ demand for innovation, improved nutritional quality, or specific dietary requirements [7,8]. In this context, combining plant-derived functional ingredients with meats other than pork has emerged as a promising strategy to address the nutritional limitations of processed meats, particularly their high saturated fat content, low fibre levels, and limited content of bioactive compounds [9,10]. These approaches are particularly advantageous in regions where local agri-food chains generate both alternative animal resources and plant-based by-products suitable for valorization.

Sicily, in southern Italy, has a long tradition of cattle farming primarily focused on milk production, alongside extensive grape cultivation, two major regional sectors supporting cheese and wine production, which are central to the local cultural heritage. Despite their importance, these sectors also generate underutilized resources: dairy-type cattle provide meat with limited commercial value [11], while the wine industry generates large amounts of grape pomace [12]. Among Sicilian cattle breeds, the Cinisara is well known for its role in the production of Caciocavallo Palermitano cheese [13,14]. However, its meat has also demonstrated good technological suitability for fermented products such as salami, owing to its high protein content and adequate intramuscular fat [15,16]. Grape pomace from Nero d’Avola grapes, one of the most widely cultivated Sicilian varieties [17], is particularly rich in polyphenols and dietary fiber [18] and is frequently used as a functional ingredient in various food matrices [19]. Despite the growing interest in plant-derived by-products and non-pork meat sources for fermented meat products, no studies have investigated the effects of GPP addition on the quality attributes of salamis produced from different dairy cattle categories.

Based on these considerations, the present study aimed to develop a novel fermented salami using meat from retired cows and young bulls of Cinisara cattle and GPP derived from the Nero d’Avola cultivar. Accordingly, the specific objectives of the study were: (i) to characterize microbial dynamics throughout salami processing using both culture-dependent and culture-independent approaches; and (ii) to evaluate the combined effects of animal category (retired cows and young bulls) and GPP inclusion on physicochemical characteristics, volatile profile, sensory traits, and consumer acceptance of salamis produced from this autochthonous dairy breed.

## 2. Materials and Methods

### 2.1. Grape Pomace Powder Production

Grape pomace from Nero d’Avola grapes was collected after post-fermentation maceration at the Cantine Europa winery (Petrosino, Trapani, Italy). Following the procedure described by Marchiani et al. [20], the pomace was dried in a natural convection chamber (Binder GmbH, Tuttlingen, Germany) at 54 °C for 48 h. It was then milled using a Retsch mill (Haan, Germany) to achieve a particle size of 250 µm. The resulting GPP was transferred into sterile PolySilk^®^ BagLight^®^ 400 bags (Interscience, Saint Nom, France) and stored at 4 °C until use.

### 2.2. Salami Production and Samples Collection

In this study, salamis were produced using meat from two categories of Cinisara cattle: four retired cows (10 years old) and four young bulls (18 months old), all reared under pasture-based management. Specifically, young bulls grazed from 6 to 16 months of age and were subsequently fed hay and concentrate from 17 to 18 months while housed in the stable, while cows were maintained predominantly on pasture with hay and concentrate supplementation until slaughter. After slaughter, the carcasses were transported to the Salumi Lipari factory in Alcamo (Trapani, Italy) and stored under refrigerated for approximately 7 days to allow ageing. Two independent salami production batches were carried out one month apart, each involving two cows and two young bulls. The carcasses were then manually deboned and trimmed to remove tendons and connective tissues. For each animal category, 16 kg of meat were individually mixed with 4 kg of pork fat from the “Suino Nero dei Nebrodi” breed which served as a standardized fat source to ensure consistent technological properties across all formulations. The mixture was minced using a grinder (Fimar 32/RS Unger, Santarcangelo di Romagna, Italy) equipped with a 6-mm plate and supplemented with 27 g/kg kitchen salt (Italkali, Palermo, Italy), 3 g/kg black pepper (Morgan Sas, Tavarnelle Val di Pesa, Italy), 0.2 g/kg sugar (Co.Pro.B., Minerbio, Italy), and 0.2 g/kg of nitrate/nitrite (Tec-Al S.r.l., Traversetolo, Italy). Each formulation was divided into two sub-batches to obtain four experimental groups (trials) differing in the presence or absence of 20 g/kg of GPP: SRC, retired cow meat without GPP; SRC-GPP, retired cow meat with GPP; SYB, young bull meat without GPP; and SYB-GPP, young bull meat with GPP. The selected inclusion level of GPP (20 g/kg) was based on preliminary trials showing that this concentration enabled the incorporation of grape pomace without compromising processing performance or overall product quality. The resulting mixtures were stuffed into natural beef casings with a diameter of 7 cm (Fortis S.r.l., Modena, Italy), using a professional sausage stuffer. All salamis underwent 6 days of drying followed by a 50-day ripening period under controlled conditions: 20–22 °C, 62–72% (relative humidity) RH at day 1; 19–21 °C, 64–74% RH at day 2; 18–20 °C, 66–76% RH at day 3; 17–19 °C, 68–78% RH at day 4; 16–18 °C, 70–80% RH at day 5; 15–17 °C, 72–82% RH at day 6; 12–14 °C, 78–88% RH from day 7 to day 22; 11–13 °C, 80–90% RH from day 23 to day 50. Samples were collected from raw materials (GPP, casings, pork fat, cow meat, and young bull meat), immediately after stuffing (T0), after drying (at day 6), and during ripening (at days 22 and 50). All samples were placed in sterile containers and transported under refrigerated conditions to the Food Microbiology Laboratories of the University of Palermo for analysis.

### 2.3. Microbiological Analyses by Culture-Dependent Approach

Twenty-five grams of each sample collected along the salami production chain were homogenized with 225 mL of buffered saline-peptone water (Condalab, Torrejón de Ardoz, Spain) using a stomacher apparatus (BagMixer^®^ 400, Interscience, Saint-Nom, France). The resulting homogenates were subsequently subjected to tenfold serial dilutions in the same isotonic solution. Aliquots of appropriate dilutions were then plated onto selective agar media for the enumeration and detection of microbial populations relevant to meat processing and fermented meat ecosystems, following the corresponding International Organization for Standardization (ISO) methods. Specifically, total mesophilic microorganisms (TMMs) [21], mesophilic lactic acid bacteria (LAB) [22], yeasts and molds [23], coagulase-positive staphylococci (CPS) and coagulase-negative staphylococci (CNS) [24], *Listeria monocytogenes* [25], members of the Enterobacteriaceae family [26], *Escherichia coli* [27], and *Salmonella* spp. [28] were enumerated. In addition, the presence of *L. monocytogenes* and *Salmonella* spp. was assessed in 25 g of GPP, raw materials, and salami samples using a two-step enrichment procedure, as described in ISO 11290-1 [29] and ISO 6579-1 [30], respectively. The limit of quantification for the enumeration method was <2 log CFU/g, while qualitative detection assays exhibited a detection limit equivalent to 0 log CFU/mL for all samples analyzed. Each analysis was performed in duplicate for all samples at each sampling point, using media and supplements purchased from Lickson (Vicari, Italy).

### 2.4. Culture-Independent Analysis of Total Bacterial Community

Total genomic DNA was extracted from GPP and salami samples immediately after stuffing and after 50 d of ripening using the DNeasy 96 PowerSoil Pro QIAcube HT Kit (QIAGEN, Hilden, Germany), following the manufacturer’s instructions. DNA purification was performed automatically on the QIAcube HT system (QIAGEN), and 5 µL of the extracted DNA was used as template for PCR amplification with primers targeting the V3–V4 region of the 16S rRNA gene [31]. PCR products were checked on a 1.5% (*w*/*v*) agarose gel, purified using Thermolabile Exonuclease I (New England Biolabs, Ipswich, MA, USA), diluted at a 1:2 ratio, and subjected to a short-cycle indexing PCR using Unique Dual Indexes (Integrated DNA Technologies, Coralville, IA, USA). Library preparation and high-throughput sequencing were performed by BMR Genomics (Padova, Italy) using the Illumina MiSeq platform (Illumina, San Diego, CA, USA) with 300 bp paired-end reads. Bioinformatic analysis was conducted using QIIME 2 version 2023.7 [32]. Raw sequences were trimmed to remove primer sequences using the q2-cutadapt plugin and denoised with q2-dada2 [33]. Taxonomic assignment of bacterial reads was performed using the Greengenes database (version 13_8), applying a 99% sequence similarity cut-off to cluster reads into Operational Taxonomic Units (OTUs). All FASTQ files generated in this study have been deposited in the Sequence Read Archive (SRA) hosted by the National Center for Biotechnology Information (NCBI) and are available under the project Ac. No. PRJNA1445583.

### 2.5. Physicochemical Analyses and Oxidant State

At each sampling time, salamis were weighed to calculate weight loss as the difference from their initial weight. In addition, pH, water activity (a_w_), and colorimetric parameters were measured in both raw materials and salami samples. The pH was measured using a digital pH meter equipped with a penetration probe (Thermo Orion 710 A+, Cambridgeshire, UK). a_w_ was measured with a dew-point hygrometer HygroLab 3 (Rotronic, Huntington, New York, NY, USA), calibrated using five saturated salt solutions with known a_w_ values. Colorimetric parameters were measured with a Chroma Meter (CR-300, Minolta, Osaka, Japan) using illuminant C, calibrated on a white standard such as L* = 100 (equivalent to BaSO4); results were expressed as lightness [L*, range from 0 (black) to 100 (white)], redness [a*, range from red (+a) to green (−a)], and yellowness [b*, range from yellow (+b) to blue (−b)], following the CIE L*a*b* system of International Commission on Illumination [34]. Hue [H = (arctg b*/a*) × 57.296] and Chroma [C = (a*^2^ + b*^2^)^0.5^] were calculated with of a* and b* values.

At the end of ripening, the hardness of fresh salamis was determined using an Instron 5564 testing machine (Instron, Trezzano sul Naviglio, Milano, Italy). Measurements were conducted on cubic samples (2 × 2 × 2 cm) equilibrated at room temperature (22 °C), and results were expressed as the maximum resistance to compression (compressive stress, N/mm^2^).

Samples of raw materials and salamis collected immediately after stuffing and at the end of ripening were immediately frozen at −20 °C, freeze-dried (SCANVAC Coolsafe 55-9, Labogene Aps, Lynge, Denmark), and then subjected to chemical analyses following standard AOAC methods [35]. These included the determination of dry matter (DM; method 967.03), ash (method 942.05), crude protein (CP; TN × 6.25, method 988.05), and ether extract (EE; method 920.29). Non-protein nitrogen (NPN) was determined according to the procedure described by Careri et al. [36] with minor modifications. Briefly, 5 g of meat were homogenized with 40 mL of distilled water for 1 min and centrifuged at 850 rpm for 15 min at 5 °C. After filtration, 20 mL of the extract were mixed with 20 mL of 5% trichloroacetic acid in water and allowed to react overnight at 4 °C. The mixture was then centrifuged and filtered, and total nitrogen (TN) in the supernatant was quantified according to AOAC method 988.05). The proteolysis index (PI) was calculated as the percentage ratio of NPN to TN.

In addition, total polyphenol content and oxidative stability were evaluated through peroxide value (POV), and thiobarbituric acid-reactive substances (TBARs) were determined. The total polyphenols were determined by the Folin–Ciocalteu colorimetric method [37] and absorbance was measured at 725 nm with a HACH DR/4000U spectrophotometer (HACH, Loveland, CO) against a reagent blank. Gallic acid aqueous solutions (0–1 mg/mL) were used to construct the calibration curve (R^2^ = 0.99), and the results were expressed as milligrams of gallic acid equivalent (GAE) per kg of sample (tq). Polyphenol content was determined only at day 0 to assess the effectiveness of GPP addition in increasing the initial phenolic content of the formulations and its potential relationship with lipid oxidation during ripening.

POV was used as an indicator of primary lipid oxidation and expressed as mEq O_2_/kg fat [38], while TBARs was used as an indicator of secondary lipid oxidation [39]. For POV determination, the calibration curve was constructed using standard iron(III) solutions in the range 0–90 µg (R^2^ = 0.99). Aliquots of 0.0, 0.1, 0.3, 0.5, 0.7 and 0.9 mL of a standard iron(III) solution (100 µg/mL in chloroform/methanol) were transferred into test tubes and brought to a final volume of 1.0 mL with chloroform/methanol (70:30, *v*/*v*). Subsequently, 25 µL of ammonium thiocyanate solution (30%, *w*/*v*) and 25 µL of hydrochloric acid (0.2 mol/L) were added. After 5 min of reaction, absorbance was measured at 500 nm, and calculations were performed according to International Dairy Federation standard [38]. TBARs values were expressed as µg of malonylaldehyde (MDA)/kg tq and quantified using a calibration curve (R^2^ = 0.99) prepared from 1,1,3,3-tetramethoxypropane solutions at different concentrations. All analyses were performed in duplicate for each independent production batch.

### 2.6. Volatile Organic Compounds Analyses

The volatile organic compound (VOC) profiles of GPP and salami samples were analyzed by solid-phase microextraction (SPME) coupled with gas chromatography–mass spectrometry (GC-MS). Briefly, 5 g of GPP and finely chopped salami were placed in sealed vials and allowed to equilibrate prior to extraction. Volatile compounds were adsorbed onto an SPME fibre (DVB/CAR/PDMS, Supelco, Bellefonte, PA, USA) at 25 °C for 60 min. Following extraction, the fibre was introduced into the GC injector and thermally desorbed at 250 °C for 5 min. Chromatographic separation was performed using a DB-624 capillary column (Agilent Technologies, Santa Clara, CA, USA; 60 m × 0.25 mm i.d., 1.40 μm film thickness). The oven temperature program started at 40 °C and was increased to 230 °C at a rate of 4 °C/min, followed by an isothermal step of 40 min and a final hold of 2 min. Mass spectrometric detection was performed in full scan mode (m/z 40–400), with the interface temperature set at 230 °C. Volatile compounds were tentatively identified by comparing their mass spectra with those in the NIST05 library. Relative abundances were calculated by expressing the peak area of each compound as a percentage of the total area of all detected peaks. All samples were analysed in duplicate for each independent production batch.

### 2.7. Sensory Evaluation

At the end of the ripening period, all salami samples were evaluated by sensory analysis conducted by a trained panel of 16 panelists (8 males and 8 females), aged between 25 and 65 years, recruited from the student, research, and teaching staff of the University of Palermo (Italy). Sensory sessions were conducted in individual tasting booths under standardized white lighting and controlled temperature conditions, in accordance with the ISO 8589 [40]. Each panelists received uniform, casing-free salami slices (approximately 5 mm thick), which were coded and served on white plates in a randomized order. Seventeen sensory descriptors, grouped into appearance, flavor, and rheological properties, were evaluated as described by Chiavari et al. [41]. Each attribute was assessed using an iPad connected to the Smart Sensory Box software (version 1.0, Smart Sensory Solutions S.r.l., Sassari, Italy). (Smart Sensory Solutions S.r.l., Sassari, Italy), and scored on a 9-point intensity scale, where 1 corresponded to the lowest perceived intensity and 9 to the highest.

### 2.8. Survey-Based Evaluation of Consumer Perception and Acceptance

Consumer perception and acceptance of novel fermented salamis produced from unconventional meats (retired cows and young bulls) and enriched with GPP were evaluated through a cross-sectional survey. The study was carried out in two retail market formats located in the province of Ragusa (Italy) and involved a total of 210 participants: 101 respondents (51.9%) at the convenience-focused store “Conad” premium supermarket and 109 respondents (48.1%) at the “Superstore Le Liccumie”. Data were collected through face-to-face interviews using a structured questionnaire. The survey design followed the methodological approach described by Garofalo et al. [42] and included questions addressing socio-demographic characteristics, consumption habits, openness to innovative food products, purchase intentions, and willingness to pay for the novel product. Table 1 summarizes the socio-demographic characteristics of the respondents, including gender, age group, educational level, household size, and annual income.

### 2.9. Statistical Analysis

Microbiological, VOCs and sensory data were analyzed using one-way analysis of variance (ANOVA). Physicochemical data were assessed through a linear mixed model, with animal category (retired cow vs. young bull) and GPP addition (with vs. without) included as fixed effects, as well as their interactions; since these were not significant, they were excluded from the models. Individual animals were included as a random effect nested within their respective animal category. Differences between treatments were evaluated separately for each ripening time, while the effect of ripening was evaluated for physical data (weight loss, pH, aw, and colorimetric data) within the same product type. In addition, a two-factor ANOVA analysis was applied to the sensory data in order to evaluate the ability of the sensory attributes to discriminate among the different salami formulations. The statistical model included panelists (i = 1, …, 16) and salami types (j = 1, …, 4) as fixed factors. Significant pairwise differences were identified using Tukey’s post hoc test, with statistical significance set at *p* < 0.05. Questionnaire response frequencies, aimed at assessing consumer perception and acceptance of the novel GPP-enriched salami, were evaluated using the Chi-square test. Statistical processing of microbiological, VOCs, sensory and questionnaire data, was carried out using XLStat (version 2020.3.1; Addinsoft, New York, NY, USA), while the physicochemical data through the SAS 9.2 software (SAS Institute Inc., Campus Drive Cary, NC, USA).

## 3. Results and Discussion

### 3.1. Microbiological Evolution

Table 2 reports the enumeration of microbial groups at each step of the salami manufacturing process, from raw materials (including GPP) to the final products after 50 days of ripening. None of the analysed samples tested positive for members of the Enterobacteriaceae family, including *E. coli* and *Salmonella* spp., nor for CPS or *L. monocytogenes*; therefore, these results, are not included in Table 2. In particular, *E. coli* and CPS counts were below 2 log CFU/g, while *Salmonella* spp. and *L. monocytogenes* were not detected in 25 g of sample, corresponding to undetectable levels (0 log CFU/g). These microorganisms are among the most relevant foodborne pathogens globally [43] and, according to Commission Regulation (EC) No 2073 [44] on microbiological criteria for foodstuffs, represent key indicators of hygiene and process control in meat products. Their absence at all processing stages indicates that raw material handling and subsequent manufacturing were conducted under conditions ensuring high hygienic standards. Notably, the microbiological analysis of GPP also revealed undetectable levels of *L. monocytogenes* and *Salmonella* spp. (0 log CFU/g), as well as counts below 2 log CFU/g for all other investigated microbial groups. This finding can be attributed to the thermal treatment applied during the drying phase [45], confirming the suitability of GPP as a safe natural ingredient for food formulations [46]. The natural beef casing exhibited a TMM count at 30 °C of approximately 4.0 log CFU/g, in agreement with values commonly reported for salted and rinsed casings [47]. Minced fat from Suino Nero dei Nebrodi, as well as meat from retired cows and young bulls, showed TMM and LAB levels exceeding 5.0 log CFU/g. Such microbial loads are expected due to the extensive surface area generated during processing [48,49], particularly as a consequence of contact with equipment and environmental surfaces [50].

Notably, the orifices of mincing machine frequently harbor high densities of LAB, which may contribute to the observed microbial counts [51]. Across all trials, regardless of GPP addition, LAB and CNS populations increased progressively during ripening, exceeding 7.0 log CFU/g after 50 d. This increase is technologically relevant, as LAB promote acidification and contribute to microbiological safety, while CNS play key roles in flavor development, color stability, and overall product quality during ripening [52,53]. Similar trends have been documented during the ripening of traditional Italian salamis [54]. Yeasts and molds, microorganisms that also contribute to aroma formation and ripening processes [55], showed a marked increase during production, reaching levels between 3 and 6 log CFU/g. Overall, these results clearly indicate that the inclusion of GPP into salami formulations did not alter the growth patterns or survival of the main microbial groups involved in fermented meat production.

### 3.2. Characterization of Salami Microbiota by Illumina Analysis

**Figure 1:**
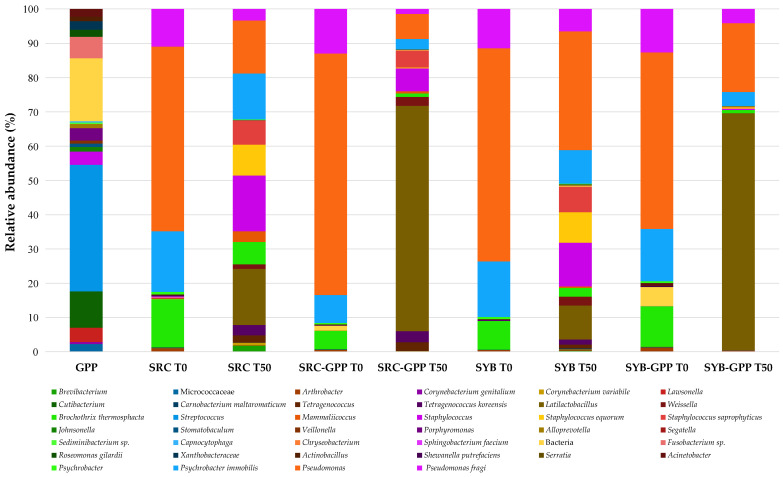
Relative abundances (%) of bacterial taxa identified in salami samples using Illumina MiSeq high-throughput sequencing. Abbreviations: GPP, grape pomace powder; SRC T0, salami production obtained from retired cow meat without GPP immediately after stuffing; SRC T50, salami production obtained from retired cow meat without GPP after 50 d of ripening; SRC-GPP T0, salami production obtained from retired cow meat with GPP immediately after stuffing; SRC-GPP T50, salami production obtained from retired cow meat with GPP after 50 d of ripening; SYB T0, salami production obtained from young bull meat without GPP immediately after stuffing; SYB T50, salami production obtained from young bull meat without GPP after 50 d of ripening; SYB-GPP T0, salami production obtained from young bull meat with GPP immediately after stuffing; SYB-GPP T50, salami production obtained from young bull meat with GPP after 50 d of ripening.

High-throughput Illumina sequencing was employed to characterize the bacterial communities of both GPP and the salami samples. This approach was adopted because it provides a comprehensive overview of food-associated microbiota, enabling the detection of both cultivable and non-cultivable taxa that are often undetected by traditional culture-dependent techniques [56]. Figure 1 reports only the OTUs with a relative abundance (RA) ≥0.1%, a threshold commonly adopted for describing microbial communities in food environments [57].

GPP showed high microbial biodiversity but was dominated by a limited number of taxa, primarily *Streptococcus*. This pattern is likely linked to processing conditions that favour more resilient taxa, as drying is known to significantly reduce LAB populations [58]. In salami samples, regardless of the animal category or GPP addition, the microbiota at the beginning of processing (T0) was dominated by spoilage-associated bacteria. In particular, *Pseudomonas* exhibited the highest RA (51.52–70.52%), followed by *Psychrobacter* (up to 17%) and *Brochothrix thermosphacta* (up to 14.0%). These bacteria are well known for their spoilage potential and their contribution to discoloration, off-odors, and textural deterioration through proteolytic and lipolytic activities [59]. Similar microbial profiles have been observed in freshly stuffed salamis produced from various animal species [7], confirming that such taxa typically dominate the early stages of sausage production. By the end of ripening (T50), these spoilage bacteria had markedly declined, while technologically relevant taxa, especially *Latilactobacillus* and *Staphylococcus* spp., increased significantly, in line with the characteristic dynamics of fermented salamis [60]. This trend was even more pronounced in GPP-enriched salamis, which showed a stronger reduction in spoilage bacteria and a notable increase in *Latilactobacillus*, reaching 65.74% RA in retired cow salamis and 69.06% in young bull salamis.

These results indicate that GPP contributes to the establishment of a selective environment that favours LAB while limiting spoilage taxa [61], thereby enhancing microbial stability and potentially improving product quality. Importantly, no major pathogenic bacteria were detected in any sample, corroborating the culture-dependent findings and confirming that all final products complied with the microbiological safety requirements established by Commission Regulation (EC) No. 2073 [44].

### 3.3. Physicochemical Analyses and Oxidant State

#### 3.3.1. Chemical Composition of Raw Materials Used for Salami Productions

Table 3 reports the chemical composition of raw materials. The water content was similar in retired cow meat (71.73%) and young bull meat (72.27%), consistent with values previously reported for Cinisara cattle raised under comparable conditions [62] and with the typical 70–75% moisture range observed in bovine meat [63]. The protein content was higher in young bull meat (20.88%) than in retired cow meat (19.39%), reflecting the greater proportion of lean muscle tissue in younger animals, as also documented in other studies on similar Cinisara categories [64].

The lipid content in both meats were relatively low, with retired cows showing a higher fat content (7.47%) than young bull (5.25%). This pattern is consistent with previous studies on pasture-based systems, where older cows generally accumulate more intramuscular fat than young bulls [15,62,64]. The lipid fraction plays a key role in oxidative stability. Pork lard, which contained 89.88% fat, represents the primary substrate for lipid oxidation and showed higher POV and TBARS values than meat, despite containing higher levels of polyphenols. These findings agree with the observations of Shahidi and Zhong [65], who reported a greater susceptibility of lipid-rich matrices to oxidative reactions.

GPP exhibited by far the highest polyphenol concentration (45.73 mg GAE/g). This winery waste is widely recognized as a rich source of bioactive phenolic compounds [66], with strong antioxidant capacity [65]. These results further support the key role of GPP in counteracting oxidative processes affecting the highly susceptible lipid fraction.

#### 3.3.2. Physical Parameters of Salamis

Table 4 reports the weight loss and physical parameters of the salamis during the ripening process. As expected, weight loss increased significantly in all samples, from approximately 10% at 6 days to more than 30% at 50 days. This trend reflects the progressive dehydration typical of dry-cured salamis [16]. No significant differences were observed among treatments, indicating that GPP addition did not influence water loss.

During the initial stages of ripening, pH showed an initial decrease followed by an increase at later stages. This trend is consistent with the typical acidification occurring during early fermentation due to LAB activity, followed by an increase commonly associated with ongoing proteolytic processes in fermented meat products [16,67]. GPP inclusion resulted in slightly higher initial pH values at 0 and 6 days. Although pH decreased during early fermentation, GPP-enriched salamis maintained slightly higher values throughout this phase. By days 22 and 50, differences among treatments were less pronounced. These observations suggest that GPP may influence early fermentation dynamics, although the underlying mechanisms were not specifically investigated in this study. These observations are consistent with literature describing potential interactions between polyphenols and microbial processes during fermentation [68].

Regarding colour parameters, lightness (L*) decreased over time, particularly in salamis produced from retired cow meat, reflecting dehydration and pigment concentration [16]. GPP-enriched salamis showed significantly lower a* (red), b* (yellow), and chroma values across all sampling times compared with their respective controls. This reduction in colour intensity may be partly attributable to a masking effect exerted by pigments naturally present in GPP and/or to changes in light scattering properties, rather than exclusively to increased pigment oxidation. During ripening, salamis without GPP showed significant reductions in a*, chroma, and hue values, indicating a decline in pigment stability. Although GPP-enriched samples exhibited lower initial colour parameters, the presence of phenolic compounds in GPP may have contributed to limiting oxidative processes affecting meat pigments [65,69,70]. Therefore, a potential role of GPP in supporting colour stability during ripening cannot be excluded, although this mechanism was not directly investigated in the present study. Finally, GPP did not affect hardness, suggesting that its inclusion does not modify the structural properties of the final product [69].

#### 3.3.3. Chemical Attributes of Salamis

Table 5 summarizes the chemical composition and oxidative status of salamis at the beginning (day 0) and at the end of ripening (day 50).

As ripening progressed, a marked decrease in water content was observed, along with a corresponding increase in proteins, lipids, and ash contents. PI increased during ripening, reflecting the enzymatic breakdown of proteins typically occurring in fermented and dry-cured meat products [16,67]. Salamis produced from retired cows and supplemented with GPP showed higher PI values than those without GPP (14.05 vs. 9.97), suggesting that the effect of GPP on proteolysis may depend on the characteristics of the raw meat. This effect may be mediated through interactions with microbial communities or proteolytic enzymes.

Regarding lipid oxidation, TBARS values increased during ripening in all samples, confirming the expected progression of oxidative reactions in cured meat products [65]. However, salamis without GPP showed higher oxidation levels in both retired cow and young bull formulations. These findings highlight the antioxidant activity of GPP, which seems to slow the formation of secondary lipid oxidation products, in agreement with previous reports on the use of natural antioxidants in meat products [69,71]. It should be noted that TBARS and POV indicate different stages of lipid oxidation: TBARS reflect the accumulation of secondary oxidation products, mainly malondialdehyde, while POV measures primary oxidation products, namely hydroperoxides. In this context, POVs followed a similar trend, although differences among treatments were less pronounced. This outcome may be attributed to the unstable and transient nature of hydroperoxides, which tend to decompose rapidly into secondary compounds during ripening, leading to more evident differences in TBARS than in POV [65].

A comparison between salamis produced from retired cows and young bulls revealed that the effect of GPP was not uniform across the two meat categories. In particular, the increase in total polyphenol content observed after GPP addition was more pronounced in salamis from retired cows than in those from young bulls. This difference may be related to variations in the chemical composition and structural properties of the raw meat, which could influence the retention, extractability, or interaction of polyphenols within the meat matrix.

### 3.4. Salami Volatilome

The volatilome was assessed in GPP before its incorporation into the formulations and in all salami samples at the end of ripening (Table 6). In GPP-free salamis, regardless of animal category, the volatile compounds identified mainly belonged to aldehydes, alcohols, acids, monoterpenes, ketones, and esters, which are chemical classes commonly reported in dry-fermented meat products [72,73].

Aldehydes represented one of the most abundant classes in both formulations, with a higher relative proportion observed in salamis produced from retired cows (30.62%) compared with those from young bulls (26.08%). This finding suggests a greater contribution of lipid oxidation pathways in salamis made from older animals, an interpretation further supported by the high levels of key oxidation markers such as hexanal, 1-penten-3-ol, and 1-octen-3-ol [74,75]. However, this trend must be interpreted in conjunction with the TBARS results, which showed higher values in the SYB samples. This apparent discrepancy may be explained by the different nature of these indicators. TBARS mainly reflect malondialdehyde formation, whereas volatile aldehydes originate from multiple lipid oxidation pathways and may follow different accumulation or degradation patterns depending on matrix interactions and fatty acid composition. Indeed, it has been reported that the evaluation of lipid oxidation based on a single marker can be misleading, as volatile compounds provide a broader representation of oxidative processes [74]. Therefore, VOC and TBARS should be considered complementary rather than overlapping indicators of lipid oxidation. Esters accounted for 8.97% of the volatile profile in salamis from retired cows and 11.11% in those from young bulls, with compounds such as ethyl propanoate and ethyl butanoate contributing fruity aromatic notes [76]. Monoterpenes were detected in both formulations and were mainly derived from spices added to the salami mixtures [73]. Ketones were present at lower RA but may nonetheless contribute to aroma complexity due to their low odor thresholds [77].

Overall, the differences observed between the two formulations indicate a slightly higher contribution of lipid oxidation to the volatile profile of salamis made from retired cows, whereas those from young bulls appear to be more strongly influenced by microbial metabolic activity. Within this context, the effects of GPP addition varied according to the type of meat used. In particular, a more pronounced increase in total polyphenol content was observed in SRC-GPP compared with SRC, whereas only a limited increase was found in SYB-GPP. A similar pattern was observed for volatile compounds, suggesting that the response to GPP incorporation may be modulated by the characteristics of the meat matrix. These differences can reasonably be associated with the intrinsic properties of the raw materials, including differences in chemical composition and oxidative status between meat from older and younger animals, which are known to affect both oxidation reactions and microbial activity [73,74].

In GPP-enriched salamis, 19 major volatile compounds were identified, predominantly belonging to alcohols, diols, and esters, highlighting the distinct contribution of GPP to the volatile profile. The incorporation of GPP significantly modified the volatilome, particularly within the ester, alcohol, and plant-derived compound classes. Ester levels increased from 8.97% to 11.30% in salamis from retired cows and from 11.11% to 13.40% in those prepared from young bull meat. This trend was further supported by the detection of compounds absent in GPP-free salamis, such as ethyl octanoate, which increased in salamis from retired cows and was newly detected in those from young bulls. Alcohols were also influenced by GPP addition: phenylethyl alcohol increased from 7.48% to 10.04% in salamis from retired cows and from 8.77% to 14.33% in those from young bulls, while 3-methyl-1-butanol (isoamyl alcohol), not detected in the control salamis, was present exclusively in GPP-enriched samples. Furthermore, the detection of plant-derived compounds such as carene and 2,3-butanediol, both absent in GPP-free salamis, confirms the direct contribution of GPP to the volatile fraction. Taken together, these findings indicate that GPP influences the volatile composition of fermented salamis primarily by introducing new aroma active compounds and inducing moderate, class-specific modifications to the existing volatile profile.

### 3.5. Sensory Evaluation of Salamis

**Figure 2:**
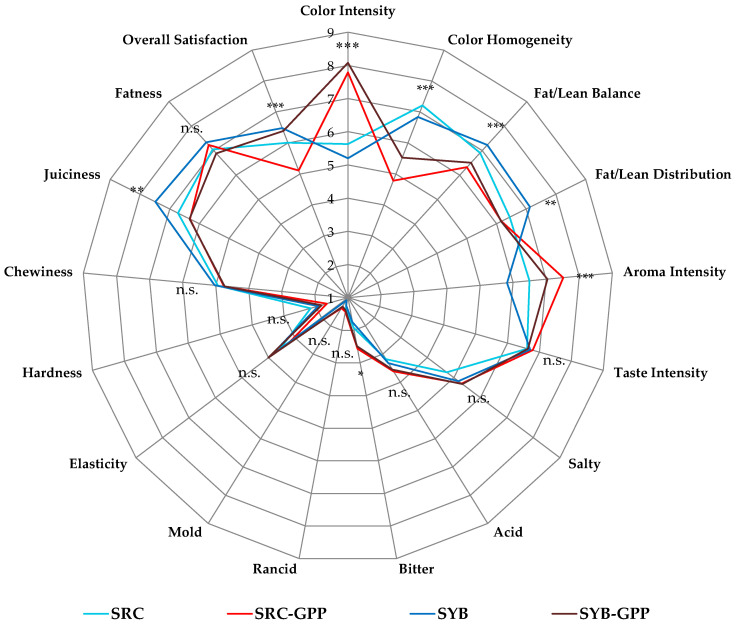
Radar chart illustrating the sensory profile of the salami samples. Abbreviations: SRC, salami production obtained from retired cow meat without grape pomace powder (GPP); SRC-GPP, salami production obtained from retired cow meat with GPP; SYB, salami production obtained from young bull meat without GPP; SYB-GPP, salami production obtained from young bull meat with GPP. * *p* < 0.05; ** *p* < 0.01; *** *p* < 0.001; n.s., not significant (*p* > 0.05).

Figure 2 presents the spider plot illustrating the sensory attributes of salamis produced in this study after 50 days of ripening. As these products may be classified as innovative fermented meat products, sensory evaluation is considered essential to assess consumer acceptability before market introduction [78]. As reported by Santhi et al. [79], the inclusion of GPP can markedly affect the sensory attributes of meat products. In this study, the addition of GPP, regardless of animal category, led to increased colour intensity, aroma intensity, as well as bitterness. These effects are largely attributable to the high content of phenolic compounds and anthocyanins in GPP [80,81]. Conversely, GPP negatively affected colour homogeneity and certain structural attributes, including the balance and distribution of fat and lean tissue. These changes are likely related to the presence of insoluble dietary fibres in GPP, which may interfere with the meat matrix structure [70]. A reduction in perceived juiciness was also noted, probably associated with modifications in water-holding capacity and textural properties [82].

Significant differences were observed among salami formulations, whereas none of the evaluated sensory attributes showed significant effects attributable to panelists (Table S1). Similar results have been reported by Bobko et al. [83] and Riazi et al. [84], who evaluated the use of red GPP in fermented and non-fermented meat products, respectively.

Overall acceptability, derived from the combined assessment of all sensory attributes [85], indicated that salamis from retired cows, both with and without GPP, were the least appreciated by panelists. This outcome is likely due to the combined effect of intense flavour, pronounced bitterness, and lower juiciness. In contrast, salamis made from young bull meat, with and without GPP, achieved comparable sensory scores, suggesting that meat from younger animals may allow a better integration of GPP without adversely affecting sensory perception. Taken together, these results suggest that the sensory impact of GPP incorporation is strongly dependent on the age and intrinsic properties of the raw meat used.

### 3.6. Consumer Perception and Acceptance of Salamis

Consumer perceptions and acceptance of the novel GPP-enriched salamis produced from dairy cattle meat were investigated through a survey conducted in two retail formats with different market positioning. These formats were deliberately selected to capture potential differences in consumer profiles between a convenience-focused supermarket and a premium retail store, in line with previous studies on consumers’ segmentation and acceptance of emerging food products and technologies [86,87].

As shown in Table 1, participants recruited at Conad were predominantly male, younger, and generally characterized by lower educational attainment. In contrast, respondents from Superstore Le Liccumie were mainly female, more frequently belonged to older age groups (45–54 and 55–64 years), and exhibited higher educational levels and household income. These findings support existing evidence that premium retail formats tend to attract more informed consumers who are more conscious of product quality and sustainability attributes, and they further highlight the role of education and income in shaping food related attitudes [88,89]. Consequently, the observed contrasts likely reflect not only structural or organizational differences between the retail environments, but also variations in the underlying sociodemographic composition of consumers. This potential confounding factor limits the extent to which the observed differences can be attributed solely to retail setting characteristics and warrants a more prudent interpretation of the results.

Table 7 summarises responses to the full set of questionnaire items. Significant differences emerged between the two retail formats with respect to familiarity with non-pork salamis (χ^2^ = 41.47, *p* < 0.0001) and familiarity with foods enriched with by-products (χ^2^ = 52.34, *p* < 0.0001).

Consumers shopping in the premium store showed markedly higher familiarity in both areas compared with those in the convenience supermarket format. These results are not surprising, as premium retail formats typically offer broader and more differentiated assortments, exposing consumers to quality-oriented and innovative products, and thereby attracting segments with distinct motivations and purchasing behaviours [90]. Moreover, consumers with higher income levels and greater health orientations are generally more accustomed to novel and functional food products [91].

Food safety perceptions were consistently high across both retail formats (χ^2^ = 1.34, *p* = 0.512), suggesting a comparable degree of trust in the safety of food products purchased in retail stores, regardless of the store type [92]. A similar pattern emerged for openness to innovation, as consumer in both groups showed favourable attitudes toward novel food concepts, including products made from unconventional meats or enriched with by-products (χ^2^ = 2.36, *p* = 0.310). This finding aligns with previous studies showing that consumers are typically receptive to food innovations when clear health, functional, or sustainability benefits are perceived [93].

Finally, in both retail formats, more than 80% of respondents indicated a willingness to pay a price premium of 10–20% compared with traditional pork salamis, demonstrating broad economic acceptance of the product. These findings reinforce the idea that, at least for certain consumer segments, perceived product quality and added value may outweigh price sensitivity in food purchasing decisions [94]. Overall, these results suggest that the novel GPP-enriched salamis have strong potential for widespread consumer acceptance across diverse retail contexts.

## 4. Conclusions

This study demonstrated that fermented salamis produced from the meat of Cinisara retired cows and young bulls can be effectively supplemented with GPP without interfering with the technological progression of spontaneous fermentation. GPP supplementation influenced several quality-related attributes, particularly oxidative stability, color evolution during ripening, volatile composition, and selected sensory properties, supporting its suitability as a functional ingredient in fermented meat products. Clear differences were observed depending on the animal category. Salamis produced from retired cow meat generally showed a higher susceptibility to lipid oxidation and more pronounced sensory modifications following GPP addition, including increased bitterness and reduced structural homogeneity. In contrast, salamis made from young bull meat exhibited a more balanced response to GPP supplementation, with better integration of the plant-derived ingredient and comparable overall sensory acceptability. These findings indicate that animal category plays a relevant role in modulating the technological and sensory effects of GPP inclusion. Although GPP supplementation proved effective in improving oxidative stability at the product level, the bioaccessibility and biological relevance of GPP-derived compounds were not evaluated in this study. Future research should therefore include larger numbers of animal carcasses, broader and more diverse consumer panels at the national scale, and the evaluation of different GPP inclusion levels. In addition, mechanistic studies, in vitro gastrointestinal digestion models, and functional antioxidant assays are warranted to better clarify the potential health-related implications of GPP incorporation into fermented meat products. Despite these limitations, the results indicate that GPP represents a promising ingredient for increasing the diversity and quality of fermented salamis produced from dairy cattle meat. This approach supports product innovation while contributing to the valorization of underutilized animal resources and winery by-products, in line with sustainability-oriented and circular economy strategies.

## Figures and Tables

**Table 1:** Socio-demographic profile of the respondent consumers (% by retail store).

Characteristic	Categories	Conad		Superstore Le Liccumie	
		Total (n)	%	Total (n)	%
Gender	Female	42	41.6	64	58.7
	Male	59	58.4	45	41.3
Age	18–24	9	8.9	8	7.3
	25–34	18	17.8	11	10.1
	35–44	23	22.8	18	16.5
	45–54	19	18.8	32	29.4
	55–64	21	20.8	36	33.0
	65+	11	10.9	4	3.7
Education	Primary school	22	21.8	13	11.9
	Secondary school	60	59.4	37	33.9
	University	19	18.8	59	54.1
Household dimension	1	23	22.8	22	20.2
	2	26	25.7	21	19.3
	3 or more	52	51.5	66	60.6
Annual income	<15,000 €	21	20.8	3	2.8
	15,000–30,000 €	42	41.6	19	17.4
	30,000–50,000 €	26	25.7	56	51.4
	>50,000 €	12	11.9	31	28.4

**Table 2:** Microbial loads of samples collected during experimental salami productions.

Samples	Bacterial Counts				
	TMMs	LAB	CNS	Yeasts	Molds
Raw materials					
GPP	<2	<1	<2	<2	<2
Casing	3.91	<1	<2	3.43	2.10
Fat	5.12	5.01	3.97	3.71	3.85
Retired cow meat	5.63	5.29	4.39	4.13	3.18
Young bull meat	5.70	5.44	4.71	3.98	2.95
Salamis at 0 d					
SRC	6.44	6.15	4.28	3.67	3.25
SRC-GPP	6.76	6.40	4.07	3.83	3.02
SYB	6.82	6.30	4.40	3.54	3.19
SYB-GPP	6.80	6.52	4.11	3.80	3.14
SEM	0.051	0.058	0.048	0.044	0.043
*p* value	0.380	0.602	0.319	0.374	0.714
Salamis at 6 d					
SRC	8.20	7.97	6.94	6.33	5.11
SRC-GPP	8.47	8.09	6.72	6.19	5.02
SYB	8.25	7.81	6.99	6.47	5.29
SYB-GPP	8.39	8.01	6.84	6.24	5.17
SEM	0.046	0.056	0.044	0.043	0.045
*p* value	0.586	0.721	0.516	0.431	0.576
Salamis at 22 d					
SRC	7.91	7.59	7.27	6.41	6.19
SRC-GPP	8.13	7.84	7.39	6.24	6.06
SYB	7.94	7.71	7.11	6.33	6.23
SYB-GPP	8.08	7.88	7.20	6.55	6.31
SEM	0.044	0.056	0.044	0.044	0.044
*p* value	0.686	0.717	0.510	0.380	0.629
Salamis at 50 d					
SRC	7.47	7.52	7.09	6.45	6.70
SRC-GPP	7.62	7.48	6.94	6.27	6.37
SYB	7.71	7.12	7.29	6.53	6.67
SYB-GPP	7.66	7.16	7.23	6.61	6.58
SEM	0.044	0.064	0.048	0.045	0.048
*p* value	0.747	0.353	0.302	0.304	0.394

Results are expressed as log CFU/g and indicate mean values of four plate counts (carried out in duplicate for two independent productions). Abbreviations: TMMs, total mesophilic microorganisms; LAB, lactic acid bacteria, CNS, coagulase negative staphylococci; GPP, grape pomace powder; SRC, salami production obtained from retired cow meat without GPP; SRC-GPP, salami production obtained from retired cow meat with GPP; SYB, salami production obtained from young bull meat without GPP; SYB-GPP, salami production obtained from young bull meat with GPP; SEM, standard error of the mean.

**Table 3:** Chemical composition of raw materials used for salami productions.

Parameters	Samples			
	GPP	Retired Cow	Young Bull	Pork Fat
Water, %	6.37 ± 0.66	71.73 ± 5.17	72.27 ± 0.84	7.49 ± 0.65
Protein, %	11.34 ± 0.16	19.39 ± 0.49	20.88 ± 0.87	2.32 ± 0.03
Fat, %	8.34 ± 0.12	7.47 ± 0.64	5.25 ± 1.65	89.88 ± 1.27
Ash, %	6.85 ± 0.10	1.07 ± 0.03	1.06 ± 0.01	0.24 ± 0.01
Polyphenols, mg GAE/g tq	45.73 ± 0.32	1.06 ± 0.06	1.12 ± 0.04	2.33 ± 0.02
POV, mEq O_2_/kg fat	n.e.	0.42 ± 0.02	0.62 ± 0.38	2.06 ± 0.02
TBARS µg MDA/kg tq	n.e.	0.23 ± 0.04	0.23 ± 0.04	1.03 ± 0.01

Results indicate mean values ± standard deviation of four determinations (carried out in duplicate for two independent productions). GPP, grape pomace powder; GAE, gallic acid equivalent; POV, peroxide value; TBARS, thiobarbituric acid-reactive substances; MDA, malondialdehyde; n.e., not evaluated.

**Table 4:** Weight loss and physical parameters of salamis.

Parameters	Ripening Time (Days)	Samples				SEM	*p* Value	
		SRC	SRC-GPP	SYB	SYB-GPP		AC	GPP
Weight loss, %	6	10.65 B	10.03 B	10.50 B	11.16 B	2.267	0.8392	0.9920
	22	21.00 AB	18.76 AB	21.06 AB	20.57 AB	2.413	0.7187	0.6005
	50	34.21 A	29.47 A	35.58 A	32.26 A	3.296	0.5626	0.2885
	SEM	2.403	2.304	2.978	3.025	n.e.	n.e.	n.e.
	*p* value	0.0141	0.0216	0.0215	0.0362	n.e.	n.e.	n.e.
pH	0	5.42 b	5.77 a	5.27 b	5.73 a	0.049	0.3846	0.0148
	6	5.44 b	5.68 a	5.35 b	5.66 a	0.010	0.0648	0.0002
	22	5.43	5.80	5.21	5.68	0.76	0.3266	0.0509
	50	5.64	6.14	5.32	5.89	0.150	0.3920	0.1475
	SEM	0.197	0.274	0.085	0.041	n.e.	n.e.	n.e.
	*p* value	0.4810	0.9280	0.3484	0.2336	n.e.	n.e.	n.e.
a_w_	0	0.88	0.92	0.90	0.95	0.011	0.1540	0.1902
	6	0.94	0.94	0.91	0.92	0.014	0.1568	0.0801
	22	0.95	0.93	0.92	0.91	0.008	0.8000	0.3920
	50	0.89	0.81	0.83	0.81	0.017	0.2211	0.3916
	SEM	0.028	0.034	0.022	0.019	n.e.	n.e.	n.e.
	*p* value	0.3712	0.1672	0.1157	0.0591	n.e.	n.e.	n.e.
Lightness L*	0	44.02 A	40.62 AB	42.24	37.28	0.965	0.2095	0.0510
	6	42.13 AB	41.98 A	40.28	38.30	0.705	0.0735	0.4639
	22	42.27 ABa	35.21 ABb	37.83 ab	36.40 b	0.665	0.2448	0.0077
	50	36.79 Bab	34.55 Bb	39.72 a	32.87 b	0.750	0.6849	0.0105
	SEM	0.666	0.869	0.832	0.736	n.e.	n.e.	n.e.
	*p* value	0.0130	0.0219	0.3529	0.1028	n.e.	n.e.	n.e.
Redness a*	0	29.18 Aa	16.25 b	29.33 Aa	18.69 b	1.361	0.6432	0.0010
	6	26.15 ABa	18.19 bc	24.21 ABac	14.20 b	0.909	0.1290	0.0003
	22	22.40 Ba	15.14 ab	21.27 ABab	14.93 b	0.889	0.7143	0.0024
	50	22.44 Ba	12.63 b	19.11 Ba	11.84	0.486	0.0560	<0.0001
	SEM	0.754	1.246	0.965	0.808	n.e.	n.e.	n.e.
	*p* value	0.0215	0.4835	0.0152	0.0679	n.e.	n.e.	n.e.
Yellowness b*	0	13.93 Aa	9.46 Ab	13.55 Aa	7.65 Ab	0.309	0.1010	<0.0001
	6	10.22 Ba	6.74 Bab	9.26 Ba	5.11 ABb	0.449	0.1136	0.0007
	22	10.03 Ba	5.71 Bb	8.03 Bab	4.63 Bb	0.444	0.1694	0.0016
	50	8.83 Ba	4.73 Bb	7.57 Ba	3.61 Bb	0.266	0.0453	<0.0001
	SEM	0.336	0.324	0.439	0.394	n.e.	n.e.	n.e.
	*p* value	0.0010	0.0014	0.0016	0.0209	n.e.	n.e.	n.e.
Chroma	0	32.34 Aa	19.86 b	32.31 Aa	20.20 b	1.039	0.9434	<0.0001
	6	28.08 Aa	19.41 bc	25.92 ABa	14.96 b	1.000	0.1243	0.0004
	22	24.57 Ba	16.18 bc	21.74 Bac	15.80 b	0.967	0.5780	0.0019
	50	24.11 Ba	13.51 b	20.57 Ba	12.39 b	0.524	0.0460	<0.0001
	SEM	0.797	0.880	1.050	0.879	n.e.	n.e.	n.e.
	*p* value	0.0115	0.0807	0.0102	0.0530	n.e.	n.e.	n.e.
Hue angle (H°)	0	25.59 A	27.02	24.80 A	22.25	1.469	0.2842	0.5346
	6	24.16 AB	20.26	20.68 B	21.25	0.557	0.2279	0.1032
	22	21.50 AB	20.46	20.53 B	18.09	0.741	0.0625	0.0578
	50	17.87 B	20.27	20.26 B	16.86	0.690	0.2647	0.0587
	SEM	0.492	1.015	0.379	0.779	n.e.	n.e.	n.e.
	*p* value	0.0233	0.2885	0.0007	0.1249	n.e.	n.e.	n.e.
Hardness, N/mm^2^	50	0.117	0.117	0.179	0.124	0.016	0.1921	0.0897

Results indicate mean values of four determinations (carried out in duplicate for two independent productions). Abbreviations: SRC, salami production obtained from retired cow meat without grape pomace powder (GPP); SRC-GPP, salami production obtained from retired cow meat with GPP; SYB, salami production obtained from young bull meat without GPP; SYB-GPP, salami production obtained from young bull meat with GPP; AC, animal category; SEM, standard error of the mean; n.e., not evaluated. Different lowercase letters (a–c) within the same row indicate significant differences among salami types (*p* < 0.05), whereas different uppercase letters (A,B) within the same column indicate significant differences within the same salami type across different ripening times (*p* < 0.05).

**Table 5:** Chemical parameters and oxidative state of salamis.

Parameters	Ripening Time (Days)	Samples				SEM	*p* Value	
		SRC	SRC-GPP	SYB	SYB-GPP		AC	GPP
Water, %	0	59.08	57.24	58.52	56.51	2.02	0.8396	0.0643
	50	38.93	42.85	35.90	39.17	1.61	0.1076	0.0531
Ash, %	0	3.50	3.76	3.86	3.74	0.12	0.3297	0.5135
	50	5.51	4.79	5.65	5.83	0.37	0.3437	0.1735
Protein, %	0	16.55	16.41	16.59	17.22	0.10	0.0503	0.1337
	50	27.11	25.53	24.37	25.80	1.05	0.4743	0.0927
Fat, %	0	20.83	22.54	20.71	21.36	2.16	0.8478	0.2249
	50	27.76	27.63	31.75	29.79	1.95	0.3738	0.2049
PI, %	0	7.65	6.75	7.93	7.91	1.18	0.7038	0.2685
	50	9.97 b	14.05 a	9.99 b	10.67 ab	0.86	0.2670	0.0441
POV, mEq O_2_/kg fat	0	2.42	1.94	2.07	1.71	0.16	0.1085	0.0691
	50	2.45	1.71	2.21	2.28	0.22	0.5640	0.2165
TBARS, µg MDA/kg tq	0	0.64	0.54	0.76	0.72	0.13	0.3880	0.6361
	50	3.27 b	2.75 c	4.85 a	3.16 bc	0.43	0.1497	0.0245
Polyphenols, mg GAE/g tq	0	1.52	1.98	1.86	1.90	0.09	0.3002	0.1151

Results indicate mean values of four determinations (carried out in duplicate for two independent productions). Abbreviations: SRC, salami production obtained from retired cow meat without (grape pomace powder) GPP; SRC-GPP, salami production obtained from retired cow meat with GPP; SYB, salami production obtained from young bull meat without GPP; SYB-GPP, salami production obtained from young bull meat with GPP; AC, animal category; PI, proteolysis index (NPN/TN ×100); POV, peroxide value; TBARS, thiobarbituric acid–reactive substances; MDA, malondialdehyde; GAE, gallic acid equivalent. On the row: a–c = *p* < 0.05.

**Table 6:** Volatile organic compounds detected in grape pomace powder and salami samples.

VOC	Samples					SEM	*p* Value
	GPP	SRC	SRC-GPP	SYB	SYB-GPP		
Aldehydes							
Propanal	n.d	4.42	3.52	5.4	4.04	0.192	0.089
Butanal	n.d	7.24 a	5.76 ab	5.89 ab	4.41 b	0.248	0.030
Hexanal	4.03	11.01 ab	11.15 a	9.12 ab	7.58 b	0.379	0.042
Octanal	n.d	2.08	1.65	1.92	1.43	0.070	0.128
Nonanal	2.02	3.66	2.91	2.34	2.50	0.131	0.060
Benzaldehyde	n.d.	1.73	1.37	1.41	1.05	0.062	0.059
Decenal	n.d.	0.48 a	0.38 a	n.d. b	n.d. b	0.047	<0.0001
Ketones							
2-Pentanone	n.d	1.04	1.62	1.41	1.05	0.064	0.053
2.3-Butanedione	n.d	1.44 ab	1.14 b	1.99 b	1.49 ab	0.077	0.024
Acetophenone	n.d	0.32 b	0.25 b	0.91 a	n.d. c	0.072	<0.0001
p-Phenylacetophenone	3.90	n.d. c	1.95 a	n.d. c	1.27 b	0.182	<0.0001
Alcohols							
2-Butanol	n.d	9.74 a	7.75 ab	8.09 ab	6.06 b	0.324	0.027
1-Penten-3-ol	n.d	5.01 a	3.99 ab	3.35 bc	2.51 c	0.248	0.007
1-Octen-3-ol	n.d	13.03 a	8.78 bc	11.26 ab	6.95 c	0.545	0.008
1-Octanol	n.d	n.d b	n.d b	2.15 a	1.61 a	0.208	<0.0001
Phenylethyl alcohol	12.30	7.48 b	10.04 b	8.77 b	14.33 a	0.606	0.007
3-Methyl-1-butanol	5.20	n.d. c	0.99 b	n.d. c	2.54 a	0.225	<0.0001
Esters							
Ethyl propanoate	n.d.	2.52 b	2.09 b	4.38 a	3.28 ab	0.201	0.003
Ethyl butanoate	n.d.	0.64 c	n.d. c	2.75 a	1.39 b	0.248	<0.0001
Methyl hexanoate	n.d.	4.71 a	2.15 c	3.98 ab	2.23 bc	0.260	0.007
Ethyl octanoate	9.05	1.10 b	3.19 a	n.d. b	2.84 a	0.284	<0.0001
Diethyl butanedioate	3.01	n.d.	n.d.	n.d.	n.d.	n.e.	n.e.
Ethyl decanoate	10.11	n.d. b	3.87 a	n.d. b	3.66 a	0.408	<0.0001
Acids							
Acetic acid	n.d	7.32	5.82	6.99	5.24	0.237	0.118
Butyric acid	n.d	6.03	4.80	5.53	4.14	0.248	0.007
Hexanoic acid	2.02	3.70 a	1.60 b	3.42 a	2.54 ab	0.193	0.007
Nonanoic acid	3.80	n.d.	n.d.	n.d.	n.d.	n.e.	n.e
4-Hydroxybutanoic acid	4.03	n.d. b	n.d. b	n.d. b	1.12 a	0.105	<0.0001
Monoterpenes							
α-Pinene	1.87	1.91 b	1.62 b	3.49 a	2.66 ab	0.167	0.003
Limonene	7.03	3.39	4.51	5.45	5.58	0.229	0.070
Carene	1.93	n.d. c	0.23 b	n.d. c	0.39 ab	0.035	0.000
Diol							
2.3-Butanediol	19.12	n.d. c	n.d. c	4.41 a	5.13 a	0.167	<0.0001
Hydrocarbons							
Heptane 2.4 dimethyl	2.70	n.d. b	1.11 a	n.d. b	1.12 a	0.121	<0.0001
Nonane	1.90	n.d. b	n.d. b	n.d. b	0.14 a	0.013	<0.0001
Nonane 2.5 methyl	2.02	n.d. b	0.63 a	n.d. b	0.44 ab	0.069	0.027
Decane	2.00	n.d.	n.d.	n.d.	n.d.	n.e.	n.e.
Dodecane	1.96	n.d.	n.d.	n.d.	n.d.	n.e.	n.e.

Data are expressed as percentages [(peak area of each compound/total peak area of significant compounds) × 100] and represent the mean of four determinations (carried out in duplicate for two independent productions). Abbreviations: GPP, grape pomace powder; SRC, salami production obtained from retired cow meat without GPP; SRC-GPP, salami production obtained from retired cow meat with GPP; SYB, salami production obtained from young bull meat without GPP; SYB-GPP, salami production obtained from young bull meat with GPP. SEM, standard error of the mean; n.d., not detected; n.e. not evaluated. On the row: a–c = *p* < 0.05.

**Table 7:** Consumer perception and acceptance variables across retail formats.

Variables	Retail Formats		χ^2^	*p* Value
	Conad	Superstore Le Liccumie		
Familiarity with salami produced from non-pork meat			41.47	<0.0001
Affirmative	13.3	56.1		
Negative	86.7	43.9		
Familiarity with foods enriched with by-products				
Yes	6.7	39.3	52.34	<0.0001
No	86.6	36.3		
I am not sure	6.7	24.4		
Perceived food safety				
Very safe	63.5	70.1	1.34	0.512
Moderately safe	23.2	17.1		
Slightly safe	13.3	12.2		
Openness to innovation				
High openness	59.1	57.4	2.36	0.310
Conditional openness with clear explanation	38.7	42.6		
No openness	2.2	0		
Willingness to pay			0.13	0.727
Affirmative	81.6	83.2		
Negative	18.4	16.8		

## Data Availability

The original contributions presented in this study are included in the article/Supplementary Material. Further inquiries can be directed to the corresponding authors.

## Supplementary Materials

The following supporting information can be downloaded at: https://www.mdpi.com/article/10.3390/foods15101792/s1, Table S1: Descriptive sensory analysis of the experimental fermented salamis.

## References

[1] Cîrstulescu A.M.A., Băilă P.A., Neagu C., Rădulescu L., Megyesi C.I. (2025). Development and characterization of a walnut enriched pork salami. J. Agroaliment. Process. Technol..

[2] Busetta G., Garofalo G., Ponte M., Barbera M., Alfonzo A., Franciosi E., Francesca N., Frusteri G., Piazzese D., Bonanno A. (2025). Replacing preservative E252 with powdered dried sumac (*Rhus coriaria* L.) fruits in “Suino Nero dei Nebrodi” salamis: Effects on microbiological, physicochemical and antioxidant properties. Food Microbiol..

[3] Grispoldi L., Ianni F., Blasi F., Pollini L., Crotti S., Cruciani D., Cossignani L. (2022). Apple pomace as valuable food ingredient for enhancing nutritional and antioxidant properties of Italian salami. Antioxidants.

[4] Toth G. (2019). Circular economy and its comparison with 14 other business sustainability movements. Resources.

[5] Pilati M. (2021). National Recovery and Resilience Plans: Empowering the Green and Digital Transitions?.

[6] Bigliardi B., Galati F. (2013). Innovation trends in the food industry: The case of functional foods. Trends Food Sci. Technol..

[7] Settanni L., Barbaccia P., Bonanno A., Ponte M., Di Gerlando R., Franciosi E., Di Grigoli A., Gaglio R. (2020). Evolution of indigenous starter microorganisms and physicochemical parameters in spontaneously fermented beef, horse, wild boar and pork salamis produced under controlled conditions. Food Microbiol..

[8] Xie P., Gao Y., Wu C., Cui B., Wang F., Wang H., Fan N. (2025). Effects of raw meat from different species on the physicochemical quality and flavor characteristics of fermented sausages. Meat Res..

[9] Alabiso M., Maniaci G., Giosuè C., Di Grigoli A., Bonanno A. (2021). Fatty acid composition of salami made by meat from different commercial categories of indigenous dairy cattle. Animals.

[10] Kausar T., Hanan E., Ayob O., Praween B., Azad Z.R.A.A. (2019). A review on functional ingredients in red meat products. Bioinformation.

[11] Berdusco N., Kelton D., Haley D., Wood K.M., Duffield T.F. (2024). Improving market (cull) dairy cows’ carcass traits and meat quality. J. Dairy Sci..

[12] Theagarajan R., Narayanaswamy M.L., Dutta S., Moses J.A., Chinnaswamy A. (2019). Valorisation of grape pomace (cv. *Muscat*) for development of functional cookies. Int. J. Food Sci. Technol..

[13] Maniaci G., Di Grigoli A., Bonanno A., Giosuè C., Ilardi V., Alabiso M. (2021). Fatty acids as biomarkers of the production season of Caciocavallo Palermitano cheese. Animals.

[14] Maniaci G., Ponte M., Giosuè C., Gannuscio R., Pipi M., Gaglio R., Busetta G., Di Grigoli A., Bonanno A., Alabiso M. (2024). Cladodes of *Opuntia ficus-indica* (L.) as a source of bioactive compounds in dairy products. J. Dairy Sci..

[15] Alabiso M., Maniaci G., Giosuè C., Gaglio R., Francesca N., Di Grigoli A., Portolano B., Bonanno A. (2020). Effect of muscle type and animal category on fatty acid composition of bresaola made from meat of Cinisara cattle: Preliminary investigation. CyTA J. Food.

[16] Gaglio R., Francesca N., Maniaci G., Corona O., Alfonzo A., Giosuè C., Di Noto A., Cardamone C., Sardina M.T., Portolano B. (2016). Valorization of indigenous dairy cattle breed through salami production. Meat Sci..

[17] Spatafora C., Barbagallo E., Amico V., Tringali C. (2013). Grape stems from Sicilian Vitis vinifera cultivars as a source of polyphenol-enriched fractions with enhanced antioxidant activity. LWT-Food Sci. Technol..

[18] Frum A., Dobrea C.M., Rus L.L., Virchea L.I., Morgovan C., Chis A.A., Arseniu A.M., Butuca A., Gligor F.G., Georgescu C. (2022). Valorization of grape pomace and berries as a new and sustainable dietary supplement: Development, characterization and antioxidant activity testing. Nutrients.

[19] García-Lomillo J., González-SanJosé M.L. (2017). Applications of wine pomace in the food industry: Approaches and functions. Compr. Rev. Food Sci. Food Saf..

[20] Marchiani R., Bertolino M., Ghirardello D., McSweeney P.L., Zeppa G. (2016). Physicochemical and nutritional qualities of grape pomace powder fortified semi-hard cheeses. J. Food Sci. Technol..

[21] (2003). Microbiology of Food and Animal Feeding Stuffs—Horizontal Method for the Enumeration of Microorganisms—Colony-Count Technique at 30 °C.

[22] (1998). Microbiology of Food and Animal Feeding Stuffs—Horizontal Method for the Enumeration of Mesophilic Lactic Acid Bacteria—Colony-Count Technique at 30 °C.

[23] (2008). Microbiology of Food and Animal Feeding Stuffs—Horizontal Method for the Enumeration of Yeasts and Moulds—Part 1: Colony-Count Technique in Products with Water Activity Greater than 0.95.

[24] (1999). Microbiology of Food and Animal Feeding Stuffs—Horizontal Method for the Enumeration of Coagulase-Positive Staphylococci (Staphylococcus aureus and Other Species)—Part 2: Technique Using Rabbit Plasma Fibrinogen Agar Medium.

[25] (2017). Microbiology of the Food Chain—Horizontal Method for the Detection and Enumeration of *Listeria monocytogenes* and of *Listeria* spp.—Part 2: Enumeration Method.

[26] (2017). Microbiology of the Food Chain—Horizontal Method for the Detection and Enumeration of Enterobacteriaceae—Part 2: Colony-Count Technique.

[27] (2005). Microbiology of Food and Animal Feeding Stuffs—Horizontal Method for the Detection and Enumeration of Presumptive Escherichia coli—Most Probable Number Technique.

[28] (2017). Microbiology of the Food Chain—Horizontal Method for the Detection, Enumeration and Serotyping of Salmonella—Part 2: Enumeration by a Miniaturized Most Probable Number Technique.

[29] (2017). Microbiology of the Food Chain—Horizontal Method for the Detection and Enumeration of *Listeria monocytogenes* and of *Listeria* spp.—Part 1: Detection Method.

[30] (2017). Microbiology of the Food Chain—Horizontal Method for the Detection, Enumeration and Serotyping of Salmonella—Part 1: Detection of Salmonella spp.

[31] Takahashi S., Tomita J., Nishioka K., Hisada T., Nishijima M. (2014). Development of a prokaryotic universal primer for simultaneous analysis of Bacteria and Archaea using next generation sequencing. PLoS ONE.

[32] Bolyen E., Rideout J.R., Dillon M.R., Bokulich N.A., Abnet C.C., Al-Ghalith G.A., Alexander H., Alm E.J., Arumugam M., Asnicar F. (2019). Reproducible, interactive, scalable and extensible microbiome data science using QIIME 2. Nat. Biotechnol..

[33] Callahan B.J., McMurdie P.J., Rosen M.J., Han A.W., Johnson A.J.A., Holmes S.P. (2016). DADA2: High-resolution sample inference from Illumina amplicon data. Nat. Methods.

[34] CIE (Commission International de l’Eclairage) (1986). Colorimetry. Volume CIE 15.2.

[35] AOAC (2012). Official Methods of Analysis of AOAC International.

[36] Careri M., Mangia A., Barbieri G., Bouoni L., Virgili R., Parolari G. (1993). Sensory property relationships to chemical data of Italian type dry cured ham. J. Food Sci..

[37] López-Andrés P., Luciano G., Vasta V., Gibson T.M., Scerra M., Biondi L., Priolo A., Mueller Harvey I. (2014). Antioxidant effects of ryegrass phenolics in lamb liver and plasma. Animal.

[38] (1991). Determination of the Peroxide Value.

[39] Botsoglou N.A., Fletouris D.J., Papageorgiou G.E., Vassilopoulos V.N., Mantis A.J., Trakatellis A.G. (1994). Rapid, sensitive and specific thiobarbituric acid method for measuring lipid peroxidation in animal tissue, food and feedstuff samples. J. Agric. Food Chem..

[40] (2007). Sensory Analysis—General Guidance for the Design of Test Rooms.

[41] Chiavari C., Coloretti F., Ferri G., Nanni M. (2007). Proposta di un metodo per l’analisi sensoriale dei salami. Ind. Aliment..

[42] Garofalo G., Pisana C., Gaglio R., Barbera M., Settanni L., Belvedere G., Marino G., Checco G.A.C., Ruta S., Caccamo M. (2026). Effect of an anti-Listeria whey protein-based edible coating activated with bacteriophage on quality attributes and consumer perception of Sicilian Canestrato Fresco cheese. Foods.

[43] Andrade A.A., Paiva A.D., Machado A.B.F. (2023). Microbiology of street food: Understanding risks to improve safety. J. Appl. Microbiol..

[44] European Commission (2005). Commission Regulation (EC) No 2073/2005 of 15 November 2005 on microbiological criteria for foodstuffs. Off. J. Eur. Union.

[45] Mainente F., Menin A., Alberton A., Zoccatelli G., Rizzi C. (2019). Evaluation of the sensory and physical properties of meat and fish derivatives containing grape pomace powders. Int. J. Food Sci. Technol..

[46] Gaglio R., Restivo I., Barbera M., Barbaccia P., Ponte M., Tesoriere L., Bonanno A., Attanzio A., Di Grigoli A., Francesca N. (2021). Effect on the antioxidant, lipoperoxyl radical scavenger capacity, nutritional, sensory and microbiological traits of an ovine stretched cheese produced with grape pomace powder addition. Antioxidants.

[47] Wysok B., Dymkowski A., Sołtysiuk M., Kobuszewska A. (2025). Assessment of microbial and heavy metal contamination of natural sheep casings from different geographic regions. Foods.

[48] Jay J.M., Loessner M.J., Golden D.A. (2005). Food protection with chemicals, and by biocontrol. Modern Food Microbiology.

[49] Doyle M.P., Diez-Gonzalez F., Hill C. (2013). Food Microbiology: Fundamentals and Frontiers.

[50] Sukumaran A.T., Holtcamp A.J., Englishbey A.K., Campbell Y.L., Kim T., Schilling M.W., Dinh T.T. (2018). Effect of deboning time on the growth of Salmonella, *E. coli*, aerobic and lactic acid bacteria during beef sausage processing and storage. Meat Sci..

[51] Francesca N., Sannino C., Moschetti G., Settanni L. (2013). Microbial characterisation of fermented meat products from the Sicilian swine breed “Suino Nero dei Nebrodi”. Ann. Microbiol..

[52] Rocchetti G., Rebecchi A., Lopez C.M., Dallolio M., Dallolio G., Trevisan M., Lucini L. (2023). Impact of axenic and mixed starter cultures on metabolomic and sensory profiles of ripened Italian salami. Food Chem..

[53] Stegmayer M.A., Sirini N.E., Ruiz M.J., Soto L.P., Zbrun M.V., Lorenzo J.M., Frizzo L.S. (2023). Effects of lactic acid bacteria and coagulase-negative staphylococci on dry fermented sausage quality and safety: Systematic review and meta-analysis. Meat Sci..

[54] Spaziani M., Del Torre M., Stecchini M.L. (2009). Changes of physicochemical, microbiological and textural properties during ripening of Italian low acid sausages: Proteolysis, sensory and volatile profiles. Meat Sci..

[55] Sidari R., Tofalo R. (2024). Dual role of yeasts and filamentous fungi in fermented sausages. Foods.

[56] Jagadeesan B., Gerner-Smidt P., Allard M.W., Leuillet S., Winkler A., Xiao Y., Grant K. (2019). The use of next generation sequencing for improving food safety: Translation into practice. Food Microbiol..

[57] Logares R., Audic S., Bass D., Bittner L., Boutte C., Christen R., Claverie J.-M., Decelle J., Dolan J.R., Dunthorn M. (2014). Patterns of rare and abundant marine microbial eukaryotes. Curr. Biol..

[58] Anghel L., Milea A.V., Constantin O.V., Barbu V., Chițescu C., Enachi E., Râpeanu G., Mocanu G.-D., Stănciuc N. (2023). Dried grape pomace with lactic acid bacteria as a potential source for probiotic and antidiabetic value-added powders. Food Chem. X.

[59] Dorn-In S., Mang S., Cosentino R.O., Schwaiger K. (2024). Changes in the microbiota from fresh to spoiled meat, determined by culture and 16S rRNA analysis. J. Food Prot..

[60] Petka K., Walczycka M. (2026). Microbiological aspects of meat fermentation: From traditional methods to advanced microflora control techniques—A systematic review. Appl. Sci..

[61] Barbaccia P., Busetta G., Barbera M., Alfonzo A., Garofalo G., Francesca N., Moscarelli A., Moschetti G., Settanni L., Gaglio R. (2022). Effect of grape pomace from red cultivar ‘Nero d’Avola’ on the microbiological, physicochemical, phenolic profile and sensory aspects of ovine Vastedda-like stretched cheese. J. Appl. Microbiol..

[62] Giosuè C., Maniaci G., Gannuscio R., Ponte M., Pipi M., Di Grigoli A., Alabiso M. (2024). Traits of mortadella from meat of different commercial categories of indigenous dairy cattle. Animals.

[63] Lawrie R.A., Ledward D.A. (2006). Lawrie’s Meat Science.

[64] Maniaci G., Alabiso M., Francesca N., Giosuè C., Di Grigoli A., Corona O., Bonanno A. (2020). Bresaola made from Cinisara cattle: Effect of muscle type and animal category on physicochemical and sensory traits. CyTA J. Food.

[65] Shahidi F., Zhong Y. (2010). Lipid oxidation and improving the oxidative stability. Chem. Soc. Rev..

[66] Beres C., Costa G.N., Cabezudo I., da Silva-James N.K., Teles A.S., Cruz A.P., Mellinger-Silva C., Tonon R.V., Cabral L.M.C., Freitas S.P. (2017). Towards integral utilization of grape pomace from winemaking process: A review. Waste Manag..

[67] Toldrà F. (2008). Dry-Cured Meat Products.

[68] Yang F., Chen C., Ni D., Yang Y., Tian J., Li Y., Wang L. (2023). Effects of fermentation on bioactivity and the composition of polyphenols contained in polyphenol-rich foods: A review. Foods.

[69] Munekata P.E., Gullón B., Pateiro M., Tomasevic I., Domínguez R., Lorenzo J.M. (2020). Natural antioxidants from seeds and their application in meat products. Antioxidants.

[70] Pereira A., Lee H.C., Lammert R., Wolberg C., Ma D., Immoos C., Casassa F., Kang I. (2022). Effects of red wine grape pomace on the quality and sensory attributes of beef hamburger patty. Int. J. Food Sci. Technol..

[71] Selani M.M., Herrero A.M., Ruiz-Capillas C. (2022). Plant antioxidants in dry fermented meat products with a healthier lipid profile. Foods.

[72] Liu Y., Cao Y., Yohannes Woldemariam K., Zhong S., Yu Q., Wang J. (2023). Antioxidant effect of yeast on lipid oxidation in salami sausage. Front. Microbiol..

[73] Lorenzo J.M., Montes R., Purriños L., Franco D. (2012). Effect of pork fat addition on the volatile compounds of foal dry cured sausage. Meat Sci..

[74] Domínguez R., Pateiro M., Gagaoua M., Barba F.J., Zhang W., Lorenzo J.M. (2019). A comprehensive review on lipid oxidation in meat and meat products. Antioxidants.

[75] Lee D., Lee H.J., Yoon J.W., Kim M., Jo C. (2021). Effect of different aging methods on the formation of aroma volatiles in beef strip loins. Foods.

[76] Lorenzo J.M., Carballo J. (2015). Changes in physico-chemical properties and volatile compounds throughout the manufacturing process of dry-cured foal sausage. Meat Sci..

[77] García González D.L., Tena N., Aparicio Ruiz R., Morales M.T. (2008). Relationship between sensory attributes and volatile compounds qualifying dry cured hams. Meat Sci..

[78] Fiorentini M., Kinchla A.J., Nolden A.A. (2020). Role of sensory evaluation in consumer acceptance of plant-based meat analogs and meat extenders: A scoping review. Foods.

[79] Santhi D., Kalaikannan A., Elango A. (2022). Functional Low-Fat Chicken Meatballs Enriched with Grape (*Vitis Vinifera*) Pomace Powder. J. Meat Sci..

[80] Delić K., Milinčić D.D., Pešić M.B., Lević S., Nedović V.A., Gancel A.L., Teissedre P.L. (2024). Grape, wine and pomace anthocyanins: Winemaking biochemical transformations, application and potential benefits. OENO One.

[81] Yu J., Ahmedna M. (2013). Functional components of grape pomace: Their composition, biological properties and potential applications. Int. J. Food Sci. Technol..

[82] Gracey P.R., Tako E. (2025). Apple and grape pomace: Emerging upcycled functional ingredients in processed meat products designed to increase polyphenol and fiber contents. Sustain. Food Technol..

[83] Bobko M., Mesárošová A., Bobková A., Demianová A., Poláková K., Švecová T., Jurčaga L. (2024). Red grape pomace addition effect on sensory properties of pork sausages. Agrobiodiversity Improv. Nutr. Health Life Qual..

[84] Riazi F., Zeynali F., Hoseini E., Behmadi H., Savadkoohi S. (2016). Oxidation phenomena and color properties of grape pomace on nitrite reduced meat emulsion systems. Meat Sci..

[85] Qasem A.A.A., Alamri M.S., Mohamed A.A., Hussain S., Mahmood K., Ibraheem M.A. (2017). Soluble fiber-fortified sponge cakes: Formulation, quality and sensory evaluation. J. Food Meas. Charact..

[86] Braghieri A., Girolami A., Riviezzi A.M., Piazzolla N., Napolitano F. (2014). Liking of traditional cheese and consumer willingness to pay. Ital. J. Anim. Sci..

[87] Testa R., Schifani G., Migliore G. (2021). Understanding consumers’ convenience orientation: An exploratory study of fresh-cut fruit in Italy. Sustainability.

[88] Gagliardi F., Brogi L., Betti G., Riccaboni A., Tozzi C. (2025). Italian consumer willingness to pay for agri-food sustainable certification labels: The role of sociodemographic factors. Sustainability.

[89] Nie W., Bo H., Liu J., Li T. (2021). Influence of loss aversion and income effect on consumer food choice for food safety and quality labels. Front. Psychol..

[90] Bonfrer A., Chintagunta P., Dhar S. (2022). Retail store formats, competition and shopper behavior: A systematic review. J. Retail..

[91] Dhakal C.K., Khadka S. (2021). Heterogeneities in consumer diet quality and health outcomes of consumers by store choice and income. Nutrients.

[92] Wu W., Zhang A., Van Klinken R.D., Schrobback P., Muller J.M. (2021). Consumer trust in food and the food system: A critical review. Foods.

[93] Laureati M., De Boni A., Saba A., Lamy E., Minervini F., Delgado A.M., Sinesio F. (2024). Determinants of consumers’ acceptance and adoption of novel food in view of more resilient and sustainable food systems in the EU: A systematic literature review. Foods.

[94] Ghazanfari S., Firoozzare A., Covino D., Boccia F., Palmieri N. (2024). Exploring factors influencing consumers’ willingness to pay healthy labeled foods at a premium price. Sustainability.

