# Supplementary material for: Red Grape Pomace as a Quality-Modulating Ingredient in Dairy Cattle Salamis
Source: Foods. 2026 May 19;15(10):1792. doi: 10.3390/foods15101792 (PMC13206027; doi:10.3390/foods15101792)
Supplement: Supplementary file 1 [file foods-15-01792-s001.zip › foods-4282424-supplementary.pdf]

Article

Red grape pomace as a quality modulating ingredient in dairy cattle salamis

Gabriele Busetta [1,†], Giuseppe Maniaci [1,†], Marcella Barbera [2], Cristina Giosuè [3,*], Simone Italia [4], Daniela Piazzese [2], Luca Settanni [1], Marco Alabiso [1] and Raimondo Gaglio [1,*]

1 Department of Agricultural, Food and Forest Sciences, University of Palermo, Viale delle Scienze, Bldg. 5, 90128, Palermo, Italy; gabriele.busetta@unipa.it (G.B.); giuseppe.maniaci@unipa.it (G.M.); luca.settanni@unipa.it (L.S.); marco.alabiso@unipa.it (M.A.)
2 Department of Earth and Marine Sciences, University of Palermo, Via Archirafi, 22, 90123, Palermo, It-aly; marcella.barbera@unipa.it (M.B); daniela.piazzese@unipa.it (D.P.)
3 Centre for Sustainability and Ecological Transition, University of Palermo, Piazza Marina, 90133, Pa-lermo, Italy
4 Le Liccumie S.r.l., Contrada Fargione Aggl. Ind., 97015, Modica, Italy; simone.italia@leliccumie.com (S.I.)
* Correspondence: cristina.giosue@cnr.it (C.G.); raimondo.gaglio@unipa.it (R.G.)
† These authors equally contributed to the work.

Table S1. Descriptive sensory analysis of the experimental fermented salamis.

Attributes	Samples				SEM	*p* value	
	SRC	SRC-GPP	SYB	SYB-GPP		Panelists	Salamis
Color Intensity	5.63 b	7.79 a	5.20 b	8.08 a	0.120	0.610	<0.0001
Color Homogeneity	7.21 a	4.78 c	6.84 a	5.53 b	0.140	0.157	<0.0001
Fat/Lean Balance	6.91 ab	6.32 b	7.23 a	6.51 b	0.162	0.207	<0.0001
Fat/Lean Distribution	6.45 b	6.17 b	7.13 a	6.16 b	0.136	0.281	<0.0001
Aroma Intensity	6.49 b	7.52 a	5.81 c	7.04 a	0.150	0.089	<0.0001
Taste Intensity	6.61	6.79	6.71	6.64	0.189	0.934	0.813
Salty	4.73	5.29	5.17	5.32	0.482	0.440	0.118
Acid	3.18	3.61	3.32	3.55	0.520	0.550	0.091
Bitter	1.92 b	2.57 a	1.75 b	2.49 a	0.126	0.091	<0.0001
Rancid	1.32	1.43	1.26	1.38	0.108	0.721	0.085
Mold	1.09	1.37	1.09	1.31	0.340	0.333	0.213
Elasticity	3.56	3.15	3.93 ±	4.00	0.411	0.120	0.067
Hardness	2.17	1.66	1.93	1.83	0.502	0.192	0.071
Chewiness	4.93	4.75	5.03	4.73	0.176	0.214	0.214
Juiciness	6.72 b	6.31 b	7.48 a	6.33 b	0.155	0.065	<0.0001
Fatness	7.05	7.23	7.34	6.89	0.390	0.480	0.082
Overall Satisfaction	6.01 b	5.11 c	6.48 a	6.39 a	0.142	0.083	<0.0001

Results indicate the mean value. Abbreviations: SRC, salami production obtained from retired cow meat without (grape pomace powder) GPP; SRC-GPP, salami production obtained from retired cow meat with GPP; SYB, salami production obtained from young bull meat without GPP; SYB-GPP, salami production obtained from young bull meat with GPP; SEM, standard error of the mean. On the row: a, b, c = $p < 0.05$.
